# Strategies to promote reporting of Surgical Care Improvement Project (SCIP) measures: a pilot survey of anesthesia department leaders

**DOI:** 10.1186/2047-0525-1-5

**Published:** 2012-07-04

**Authors:** Rebecca M Speck, Mark D Neuman, Andrew R Bond, Lee A Fleisher

**Affiliations:** 1 Department of Anesthesiology and Critical Care, University of Pennsylvania, 3400 Spruce Street, Philadelphia, PA, 19104, USA; 2 Department of Biostatistics and Epidemiology, University of Pennsylvania, 423 Guardian Drive, Blockley Hall, Philadelphia, PA, 19104-6021, USA; 3 Department of Anesthesia, Brigham and Women’s Hospital, 75 Francis Street, Boston, MA, 02115, USA

**Keywords:** Performance measures, Surgical Care Improvement Project

## Abstract

**Background:**

The Surgical Care Improvement Project (SCIP) is a quality improvement initiative focused on reducing surgical complications. Reporting SCIP performance measures helps determine whether hospitals receive the full payment update from the Centers for Medicare and Medicaid Services. Strategies in use by hospitals to motivate departmental participation in SCIP reporting are poorly understood.

**Methods:**

A 12-item pilot survey exploring strategies to promote reporting of SCIP measures was developed and mailed to department of anesthesiology chairs at 1,426 US hospitals. Descriptive statistics and *χ*^2^ analysis were used to summarize respondent and survey data.

**Results:**

In all, 29.5% of the sample responded to the survey, with 96.9% indicating SCIP participation; 62.5% participated primarily for voluntary reasons, and 4.2% reported an incentive from their hospital as the primary reason for participation.

**Conclusions:**

Hospital strategies promoting physician participation in SCIP currently vary. A minority of survey respondents indicated that an incentive was used to encourage adherence to SCIP measures. Further research to optimize such strategies may support future efforts to improve perioperative care.

## Background

In recent years, the use of financial incentives to drive improvements in the quality of health care has garnered significant attention in the US and abroad [1-3]. The Surgical Care Improvement Project (SCIP), initiated on 1 July 2006 [4], provides a mechanism for hospitals to receive financial incentives for efforts to reduce surgical complications, such as surgical site infections, perioperative myocardial infarction, and venous thromboembolism, through improved processes of perioperative care [5]. Current SCIP measures relate to the appropriate use of preoperative antibiotics (selection, timing, and discontinuation), routine venous thromboembolism prophylaxis (ordering and administration), appropriate hair removal practices, perioperative use of beta-blockers, and perioperative normothermia and normoglycemia in selected patients.

Medicare payment rules create financial incentives for hospitals to report data on SCIP measures, as hospitals that do not submit data on selected measures are subject to Medicare reimbursement reduction [6]. Per the Deficit Reduction Act of 2005, a payment reduction of 2.0 percentage points is implemented for hospitals that fail to report successfully [7]. According to the Centers for Medicare and Medicaid Services (CMS), as of March 2009, nearly 3,700 hospitals reported performance of SCIP measures [8], with only 30 general acute-care hospitals nationwide abstaining from collection and reporting of such data [9]. Of participating facilities, 97% have received the full annual payment incentive from Medicare each year [7].

Despite high rates of participation in SCIP, little is known regarding the mechanisms hospitals may employ to promote physician participation in reporting. Multiple potential strategies exist for a hospital to encourage better practices at the level of the individual provider, including the re-engineering of clinical systems [10], provision of feedback or education to care providers [11], and creation of department-level or physician-level incentives for improved performance [12].

Efforts to understand the range of strategies used to promote physician adherence to reporting performance measures within US hospitals have implications for health policy, potentially offering insights to inform the design and dissemination of future quality improvement initiatives. To explore hospital strategies to promote anesthesiologist participation in SCIP, a pilot survey of anesthesiology department administrators at general acute-care hospitals in the US was undertaken. Respondents were surveyed on a range of hospital-level and department-level variables and on participation in SCIP, focusing on reasons for participation in the reporting of a SCIP measure relating to the dosing of preoperative antibiotics.

## Methods

After obtaining approval from the University of Pennsylvania Institutional Review Board, a multiple-choice questionnaire was developed based on literature review, clinical experience, and consultation with experts in quality improvement. The final 12-item survey instrument included items relating to hospital characteristics (hospital affiliation, urban/rural location, state, bed number), anesthesia department business model (private practice, hospital employed, academic group practices, Department of Veterans Affairs, other governmental agency), participation in SCIP reporting, hospital practices for ordering of preoperative antibiotics, hospital practices for administration of preoperative antibiotics, and participation in pay-for-performance measures.

Respondents were offered four common reasons for SCIP participation, focusing on SCIP-INF-1, a measure relating to perioperative antibiotic administration; respondents indicated one ‘primary’ and one or more ‘secondary’ reasons for participation. The four reasons for SCIP participation were: (1) participation as part of a voluntary quality improvement initiative, (2) participation in preparation for pay-for-performance, (3) participation as mandated by the anesthesia department’s service contract, and (4) participation in response to an incentive from the hospital.

Hospital data from the 2005 American Hospital Association (AHA) survey was used to identify hospitals of comparable size and to obtain hospital addresses. Our survey sample was restricted to acute-care facilities with at least 200 beds, yielding a total survey sample of 1,426 hospitals. The 12-item questionnaire was distributed through a single mailing via first-class US mail to all hospitals identified in our study population and was addressed to ‘Chair, Department of Anesthesiology’. Completed questionnaires were returned via facsimile; no monetary or material incentive was provided to survey subjects. All questionnaires were mailed in February 2008; a period of four months was allowed for survey responses.

One investigator entered all study data. The full 2006 AHA sample, the most recent available data at the time of analysis, was used as a comparison group for pilot survey respondents. The survey respondents and AHA sample were compared on US Census region, hospital bed size, rural status, and hospital ownership using Pearson’s *χ*^2^ test. To create categories comparable to those used in the AHA survey, survey respondents indicating their ownership status as ‘University Affiliated’ were grouped with private, not-for-profit facilities. Descriptive statistics determined the proportion reporting SCIP participation and the distribution of department business models identified by survey respondents. The distribution of reported reasons for SCIP participation was determined. All analyses were conducted using Stata 10.0 Software (StataCorp, College Station, TX, USA).

## Results

Questionnaires were mailed to 1,426 US hospitals. Completed questionnaires were received from 421 (29.5%) of the study sample. Table 1 displays characteristics reported by survey respondents. Of all survey respondents, the largest proportion worked in hospitals located in the Northeastern USA (33.9%). The *χ*^2^ analysis revealed significant differences between the present survey respondents and all US hospitals with 200 or more beds, as identified in the 2006 AHA survey. Overall, AHA survey data indicated a greater proportion of hospitals located in the Southern USA (38.7%) and fewer in the Northeast (20.9%) than the respondents to the present survey (*P* <0.0001). The majority of respondents to the present survey reported working in a facility with 200 to 500 beds (76.6%), which did not differ significantly from respondents to the 2006 AHA survey (80.7%) (*P* = 0.07). The fraction of rural hospitals differed significantly from the AHA sample, in which only 1.2% of hospitals were located in rural areas (*P* <0.0001).

**Table 1:** Descriptive characteristics of survey respondents

	**Respondents, N (%)**	**2006 AHA sample, N (%)**	***P*** **value**
SCIP participation	408 (96.9%)	-	
Region			<0.0001
Northeast	140 (33.9)	296 (20.9)	
South	117 (28.3)	548 (38.7)	
Midwest	93 (22.5)	317 (22.4)	
West	63 (15.2)	254 (18.0)	
Hospital location			<0.0001
Rural	47 (11.7)	17 (1.2)	
Hospital ownership			0.003
Non-profit*	316 (75.2)	1,003 (70.0)	
For profit	45 (10.7)	177 (12.4)	
VA	16 (3.8)	63 (4.4)	
Other governmental	43 (10.2)	189 (13.2)	
Hospital size			0.07
Over 500 beds	97 (23.4)	277 (19.3)	
Under 500 beds	318 (76.6)	1,155 (80.7)	

*Includes 86 respondents indicating ‘university-affiliated hospital’. SCIP, Surgical Care Improvement Project.

In terms of hospital ownership, survey respondents worked primarily at private, not-for-profit hospitals (n = 230, 54.8%) and university-affiliated hospitals (n = 86, 20.5%). Non-profit status was not specifically indicated by all who identified as a ‘university-affiliated hospital’, however, those facilities were considered to be non-profit entities for comparison with the 2006 AHA survey. The *χ*^2^ analysis revealed significant differences between the distribution of our study sample and that of the 2006 AHA survey sample in regards to hospital ownership (*P* = 0.003).

The business model for the majority of respondents was a private practice group (64.4%). Fewer reported working directly for a hospital (12.4%), an academic group practice separate from a medical school or health system (11.6%), an academic group practice within a medical school or health system (5.2%), an independent contractor (1.9%), the US Department of Veterans Affairs (3.6%), or the US Military or Public Health Service (0.7%).

Most respondents participated in SCIP reporting for preoperative antibiotic administration (n = 408, 96.9%). Table 2 lists the reasons indicated for SCIP participation. The majority of respondents (62.5%) indicated that participation in SCIP occurred for primarily voluntary reasons. A minority indicated an incentive from the hospital (4.2%) or a contractual mandate (2.5%) as the primary reason for participation. Considering primary and secondary reasons for SCIP participation, the proportions indicating a hospital incentive or a contractual mandate rose to 14.7% and 5.9%, respectively. Approximately one-fifth (20.2%) of respondents did not select a primary or secondary reason for SCIP participation.

**Table 2:** Reasons for participation in the Surgical Care Improvement Project (SCIP)*

**Reasons for SCIP participation**	**Primary reason**		**All reasons**	
	**N (%)**	**95% CI**	**N (%)**	**95% CI**
Part of a quality improvement initiative- voluntary	255 (62.5)	57.8 to 67.2	299 (73.3)	69.0 to 77.6
Mandated by anesthesia service contract	10 (2.5)	0.9 to 3.9	24 (5.9)	3.6 to 8.2
Preparation for pay-for performance	37 (9.1)	6.3 to 11.9	171(41.9)	3.7 to 4.7
Incentive from hospital for participation	17(4.2)	2.2 to 6.1	60 (14.7)	11.3 to 18.2

*20.2% (85) of respondents provided no reason, and 21.8% (89) of respondents provided no primary reason. CI, confidence interval.

## Discussion

Defining the strategies employed by hospitals to encourage adherence to reporting performance of quality measures is of relevance to current and planned efforts to improve perioperative care. CMS’s ongoing initiative to link payment to SCIP reporting [13], its planned expansion to include additional SCIP measures for 2010 and 2011 [7], and the proposed role of SCIP measures as a model for a Medicare pay-for-performance program [14,15], all suggest that reporting of such measures will continue to grow in importance as a part of the structure of reimbursement for perioperative care in the US. As a result, policymakers and hospital administrators will have a growing need for information describing optimal strategies to encourage SCIP participation across a range of hospitals.

This pilot study of 1,426 anesthesia department chairs suggests that strategies to promote SCIP participation among anesthesiologists vary among hospitals. While the majority of our 421 respondents indicated voluntary participation in SCIP, we observed that a minority reported incentives or contractual mandates as primary or secondary reasons for SCIP participation. At these facilities, it appears likely that SCIP participation has been achieved without use of financial or other incentives (or contractual mandates) for the clinicians providing the data; while we did not collect data on actual adherence to SCIP measures, this finding offers a preliminary suggestion that anesthesiologists may be willing to participate in quality improvement initiatives on a voluntary basis.

These results should be interpreted in the context of multiple limitations. Our 29.5% response rate, combined with differences noted between respondent hospitals and those in the US at large, as indicated by the 2006 AHA survey, limits the degree to which our findings can be generalized to US hospitals at large. Eligible participants only received one mailing and no follow-up was completed. Further, as the study sample was constrained to hospitals over 200 beds our findings may not be applicable to smaller hospitals. We specified four potential reasons for SCIP participation *a priori*, yet some respondents likely participated in SCIP for other reasons, which we were unable to assess through the present survey instrument. Roughly 20% of survey respondents failed to provide a reason for their participation, which could indicate either the reason they participated was not offered as an answer option or that they did not know why they participated, limiting our ability to assess our principal hypothesis. Allowing respondents an answer option of ‘other, please explain’ would have given participants the opportunity to provide their alternative reasoning.

Further, although it cannot be determined from the present survey, the possibility exists that responses may have been influenced by the existence of a similar quality-reporting program whose goals overlap with those of SCIP. Specifically, the Physician Quality Reporting System (PQRS), Medicare’s pay-for-reporting initiative, includes measures related to antibiotic dosing [16]; thus, participation in PQRS could have been conceivably confused by survey respondents for SCIP participation. Both SCIP and PQRS are measures of quality compliance with overlap in multiple content areas. The key difference is that in PQRS, financial incentives are targeted at individual physicians or physician practices, while SCIP offers incentives to hospitals. Whether there was any confusion, given the similarities of these two measures, is unknown. Lastly, as we were unable to confirm the identity of the individual completing the survey, we have limited insight into the degree to which survey responses reflect actual hospital practices.

Despite these limitations, our findings have relevance to current and planned perioperative quality improvement efforts. As regulators and payers seek to promote improvements in the quality of hospital care through an increasing number of reportable quality measures [7], effective implementation of such quality improvement initiatives will require an understanding of the considerations affecting individual hospitals’ efforts to encourage individual physicians.

## Conclusions

Our study offers preliminary insight into the range of strategies now in use in US hospitals to encourage physician participation in SCIP. The results of this pilot study suggest future hypotheses for exploration. For example, though we found high rates of departmental SCIP participation, we did not collect data on rates of adherence to specific SCIP measures, or the types of efforts departments engaged in to collect and report data to SCIP. Understanding variations in the mechanics of data collection and reporting may hold potential benefits by highlighting practices and procedures for data collection that may be more or less efficient and accurate than others. Further, it would be of interest to determine whether a direct feedback of that data, built into the data collection system, would motivate providers to improve quality. We encourage further research to support hospital administrators and quality advocates in improving the care delivered in the perioperative period.

## Competing interests

The authors declare that they have no competing interests.

## Authors’ contributions

RS was involved with data analysis, manuscript preparation, and approval of the final manuscript. MN was involved with data analysis, manuscript preparation, review of original study data and data analysis, and approval of the final manuscript. AB was involved with study design, conduct of study, and approval of the final manuscript. LF was involved with study design, conduct of study, manuscript preparation, review of original study data and data analysis, and approval of the final manuscript. All authors read and approved the final manuscript.
